# Preoperative nomogram for predicting spread through air spaces in clinical-stage IA non-small cell lung cancer using ^18^F-fluorodeoxyglucose positron emission tomography/computed tomography

**DOI:** 10.1007/s00432-024-05674-w

**Published:** 2024-04-10

**Authors:** Yun Wang, Deng Lyu, Chao Cheng, Taohu Zhou, Wenting Tu, Yi Xiao, Changjing Zuo, Li Fan, Shiyuan Liu

**Affiliations:** 1 Department of Radiology, Second Affiliated Hospital of Navy Medical University, 415 Fengyang Road, Huangpu District, Shanghai, 200003 China; 2 Department of Nuclear Medicine, Changhai Hospital, Navy Medical University, Shanghai, 200433 China

**Keywords:** Spread through air spaces, Nomogram, ^18^F-FDG PET/CT, Non-small cell lung cancer

## Abstract

**Purpose:**

This study aims to assess the predictive value of ^18^F-fluorodeoxyglucose positron emission tomography/computed tomography (^18^F-FDG PET/CT) radiological features and the maximum standardized uptake value (SUV_max_) in determining the presence of spread through air spaces (STAS) in clinical-stage IA non-small cell lung cancer (NSCLC).

**Methods:**

A retrospective analysis was conducted on 180 cases of NSCLC with postoperative pathological assessment of STAS status, spanning from September 2019 to September 2023. Of these, 116 cases from hospital one comprised the training set, while 64 cases from hospital two formed the testing set. The clinical information, tumor SUV_max_, and 13 related CT features were analyzed. Subgroup analysis was carried out based on tumor density type. In the training set, univariable and multivariable logistic regression analyses were employed to identify the most significant variables. A multivariable logistic regression model was constructed and the corresponding nomogram was developed to predict STAS in NSCLC, and its diagnostic efficacy was evaluated in the testing set.

**Results:**

SUV_max_, consolidation-to-tumor ratio (CTR), and lobulation sign emerged as the best combination of variables for predicting STAS in NSCLC. Among these, SUV_max_ and CTR were identified as independent predictors for STAS prediction. The constructed prediction model demonstrated area under the curve (AUC) values of 0.796 and 0.821 in the training and testing sets, respectively. Subgroup analysis revealed a 2.69 times higher STAS-positive rate in solid nodules compared to part-solid nodules. SUV_max_ was an independent predictor for predicting STAS in solid nodular NSCLC, while CTR and an emphysema background were independent predictors for STAS in part-solid nodular NSCLC.

**Conclusion:**

Our nomogram based on preoperative ^18^F-FDG PET/CT radiological features and SUV_max_ effectively predicts STAS status in clinical-stage IA NSCLC. Furthermore, our study highlights that metabolic parameters and CT variables associated with STAS differ between solid and part-solid nodular NSCLC.

**Supplementary Information:**

The online version contains supplementary material available at 10.1007/s00432-024-05674-w.

## Introduction

According to the 2023 Burden of Cancer Disease Report in China, lung cancer remains one of the malignant tumors with the highest incidence and mortality rates (Qi et al. 2023). In 2015, the World Health Organization (WHO) officially recognized spread through air spaces (STAS) as the fourth invasive pattern of lung adenocarcinoma, in addition to non-lepidic growth, stromal infiltration, vascular and pleural infiltration. STAS is defined as tumor cells extending beyond the tumor edge within the lung parenchyma (Travis et al. 2015; Nicholson et al. 2022), which can be observed in other type of NSCLC, such as squamous cell carcinoma, lung pleomorphic carcinoma, and lung neuroendocrine tumors (Lu et al. 2017; Yokoyama et al. 2018; Aly et al. 2019). The presence of STAS indicated the presence of potential residual tumor cells in the surgical margins of patients who underwent limited resection such as segmentectomy or wedge resection, resulting in a poorer prognosis (Kadota et al. 2015; Eguchi et al. 2019; Bains et al. 2019; Huang et al. 2022; Ren et al. 2019). More recently, in a meta-analysis to compare prognostic outcomes between lobectomy and sublobar resection in stage I NSCLC patients with STAS, the results indicated that sublobar resection resulted in worse outcomes than lobectomy in stage I NSCLC patients with STAS (Li et al. 2022a, b). Therefore, lobectomy seems to be effective for stage I NSCLC patients with STAS and a comprehensive understanding of STAS is imperative for the judicious selection of suitable surgical modalities.

In fact, a recent report demonstrated negative predictive value of STAS diagnosis on frozen section was extremely low in 48 cases of resected lung adenocarcinoma, even though the diagnostic sensitivity and specificity of STAS on frozen section were 50% and 100% (Walts et al. 2018). It should be noted that there is insufficient published data to support intraoperative detection of STAS.

^18^F-FDG PET/CT, as a noninvasive imaging method, can simultaneously depict the morphological characteristics and glucose metabolic activity of tumors, exhibiting high accuracy in predicting tumor invasion, lymph node metastasis, and visceral pleural invasion (Volpi et al. 2018; Li et al. 2022a, b; Nakanishii et al. 2021; Wang et al. 2023). Previous studies have indicated correlations between certain CT features of lung cancer and STAS status, offering a potential means of predicting STAS before surgery (Yin et al. 2020). Wang et al. (2020) demonstrated the value of SUV_max_ and total lesion glycolysis (TLG) as indicators for predicting STAS, achieving a diagnostic accuracy of 71.1% and an area under the curve (AUC) value of 0.759. Nishimori et al. (2022) identified an SUV_max_ cut-off value of 2.48, yielding a sensitivity, specificity, and AUC value of 89.5%, 52.8%, and 0.74, respectively. Few studies have explored predicting STAS based on ^18^F-FDG PET/CT metabolic parameters combined with CT features. The aforementioned studies mainly focused on lung adenocarcinoma, employed variables only based on preoperative ^18^F-FDG PET/CT metabolic parameters, featured small sample sizes, and lacked external validation.

As far as we know, no previous study on STAS has differentiated between clinical-stage IA NSCLC manifesting as solid or sub-solid nodules at ^18^F-FDG PET/CT. The SUVmax value and CT presentations reflect different levels of histologic aggressiveness within the clinical-stage IA NSCLC spectrum. As a result, it is conceivable that they also have different incidences and preoperative ^18^F-FDG PET/CT patterns that affect STAS. Therefore, we sought to analyze the incidence and ^18^F-FDG PET/CT patterns of STAS in clinical-stage IA NSCLC on the basis of their CT presentation as solid or sub-solid nodules.

This study focused on clinical-stage IA non-small cell lung cancer (NSCLC), excluding pure ground glass nodular (pGGN) lung cancer, which does not exhibit STAS (Kim et al. 2018). Our primary aim was to investigate the risk predictors associated with STAS in this type of lung cancer based on ^18^F-FDG PET/CT radiological features and SUV_max_ and guide clinical treatment selection and prognosis evaluation while exploring potential differences in the risk variables predicting STAS status in tumors with different densities.

## Materials and methods

### Patients

**Fig. 1:**
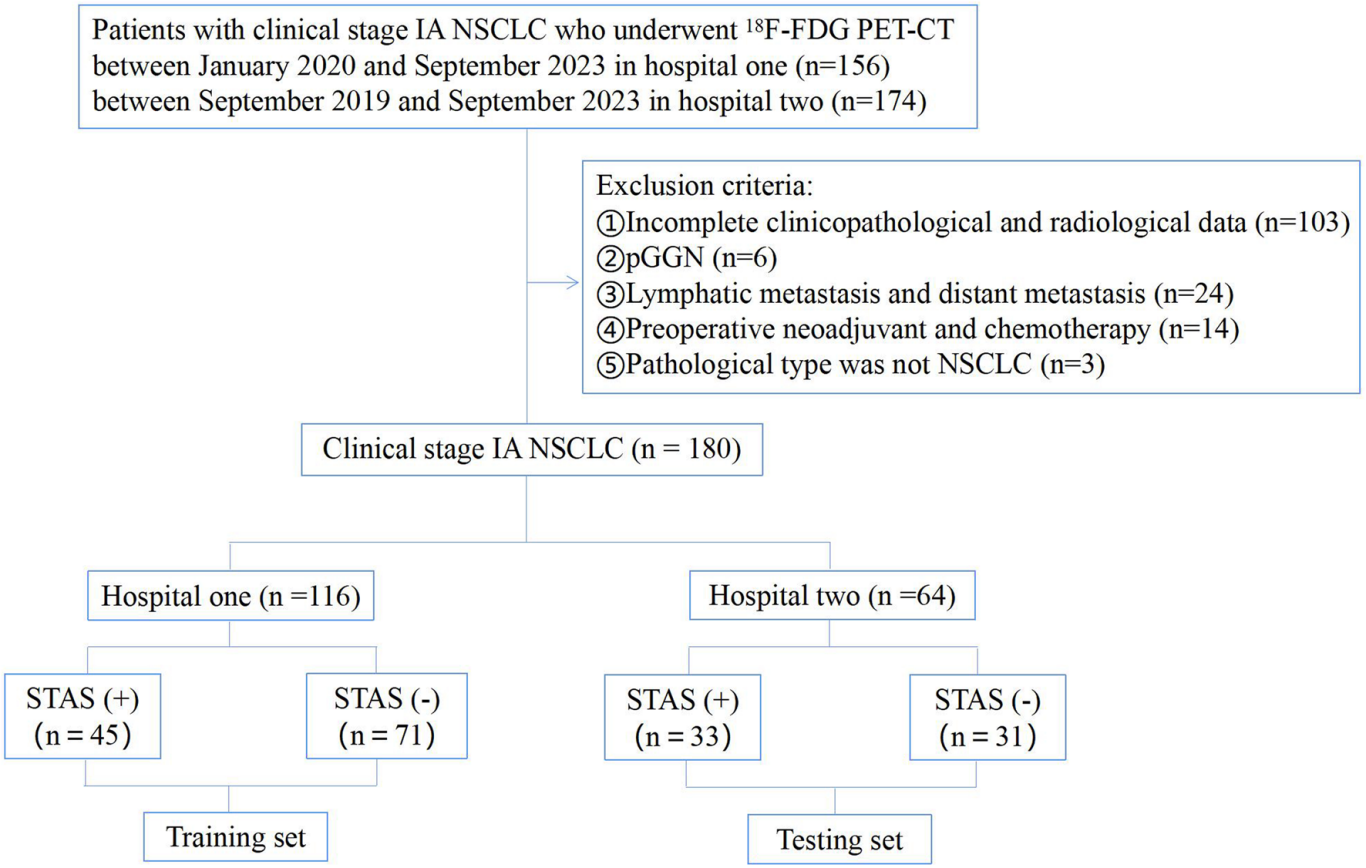
The flowchart of inclusion and exclusion criteria for the cases

Patients diagnosed with clinical-stage IA NSCLC who underwent ^18^F-FDG PET/CT examination and postoperative pathological evaluation of STAS status were retrospectively analyzed in two hospitals, spanning from September 2019 to September 2023. Clinicopathological data, including gender, age, smoking status, carcinoembryonic antigen levels (CEA), surgical type, lymph node metastasis, and pathological type, were collected for each patient. The inclusion criteria comprised: (1) ^18^F-FDG PET/CT data obtained within 2 weeks before surgery; (2) clinical-stage IA NSCLC; (3) solid or part-solid nodules in CT presentation; (4) pathological confirmation of NSCLC. Exclusion criteria encompassed: (1) incomplete clinicopathological and radiological data; (2) pGGN; (3) evidence of pathological lymph node or distant metastasis; (4) receipt of preoperative neoadjuvant or chemotherapy; (5) pathological type was not NSCLC. Based on postoperative pathology results, patients were categorized into STAS-positive and STAS-negative groups. The training set comprised 116 cases (45 STAS-positive and 71 STAS-negative) from hospital one, while the testing set included 64 cases (33 STAS-positive and 31 STAS-negative) from hospital two. Figure 1 illustrates the inclusion and exclusion criteria for the cases. In cases where a single patient presents with multiple nodulars meeting the inclusion criteria, we followed the methodology outlined by Dercle et al. (Dercle et al. 2020) to designate a representative nodular with the largest diameter for retrospective analysis.

This study received approval from the Ethics Committee of hospital one (decision number: CZ-20220712-03), and the subjects’ informed consent was waived.

### Equipment and parameters

Both the training and testing set underwent whole-body PET/CT tomography utilizing the Siemens Biograph Truepoint64 PET/CT system from Germany. The ^18^F-FDG used for the scans was supplied by the same company, ensuring a radiochemical purity exceeding 95%. For detailed scanning methods and parameters, refer to Supplemental Content 1.

### Image measurement and evaluation

The DICOM images from the patient’s ^18^F-FDG PET/CT scans were imported into Radiant DICOM Viewer software (Version 4.2.1, Medixant, Poland). To display the CT features of the lesions, multi-planar reconstruction (MPR) and maximum intensity projection (MIP) techniques were employed. For quantitative parameter evaluation, two radiologists, each with 7 and 10 years of experience, measured the lesions. The average of their measurements was considered the final result. CT quantitative features included the maximum diameter of the tumor and the maximum diameter of solid components, measured on MPR high-resolution computed tomography (HRCT) lung window images. The consolidation-to-tumor ratio (CTR) was calculated (Suzuki et al. 2011). In terms of measuring metabolic parameters, a region of interest (ROI) covering the tumor was set layer by layer on ^18^F-FDG PET/CT fusion images, non-tumor areas were avoided as much as possible. The SUV_max_ value of each slice was automatically generated, and the maximum value from all slices was considered the SUV_max_ value of the tumor. For qualitative features evaluation, two radiologists with 7 and 10 years of experience, respectively, interpreted the CT features of the lesions using a double-blind method. Consensus was reached through consultation in case of differing opinions. HRCT lung window observation included the assessment of tumor density type (solid, part-solid), margin characteristics (lobulation, spiculation), interface definition (well-defined, ill-defined), adjacent features (pleural indentation, vascular convergence, bronchial change), emphysema background, and the presence of Ground glass ribbon sign. Definitions of CT features can be found in Supplemental Table 1.

**Table 1:** Clinical and pathological datas of patients in the training and testing set

Variables	Training set (*n* = 116)			Testing set (*n* = 64)		
	STAS (-) (*n* = 71)	STAS ( +) (*n* = 45)	*P* value	STAS (-) (*n* = 31)	STAS ( +) (*n* = 33)	*P* value
Gender			0.236^a^			0.083^a^
Female	38 (53.5%)	19 (42.2%)		17 (54.8%)	11 (33.3%)	
Male	33 (46.5%)	26 (57.8%)		14 (45.2%)	22 (66.7%)	
Age (year)	64 (58.0,68.0)	65 (57.5,72.0)	0.322^c^	63.1 ± 8.9	62.6 ± 9.0	0.816^d^
Smoking status			0.359^a^			0.196^a^
Non-smoker	50 (70.4%)	28 (62.2%)		20 (64.5%)	16 (48.5%)	
Smoker	21 (29.6%)	17 (37.8%)		11 (35.5%)	17 (51.5%)	
CEA (μg/L)			0.216^a^			0.092^a^
< 5	64 (90.1%)	37 (82.2%)		27 (87.1%)	23 (69.7%)	
≥ 5	7 (9.9%)	8 (17.8%)		4 (12.9%)	10 (30.3%)	
Surgery type			0.407^a^			1.000^b^
Sublobectomy	19 (26.8%)	9 (20.0%)		5 (16.1%)	5 (15.2%)	
Lobectomy	52 (73.2%)	36 (80.0%)		26 (83.9%)	28 (84.8%)	
Pathological type			0.037^a^			0.281^a^
Adenocarcinoma	66 (93.0%)	36 (80.0%)		26 (83.9%)	24 (72.7%)	
Non-adenocarcinoma	5 (7.0%)	9 (20.0%)		5 (16.1%)	9 (27.3%)	

Note: a, Pearson’s Chi-square; b, Yate’s correction for continuity; c, the Mann–Whitney U test; d, the two independent samples *t* test; STAS, spread through air spaces; CEA, carcinoembryonic antigen

### Pathological diagnosis

The diagnosis of STAS was established using the pathological reports obtained from two hospitals. The diagnostic criteria outlined by the WHO in 2015 were uniformly applied to assess STAS status in both medical facilities (Travis et al. 2015). The evaluation involved the examination of at least two hematoxylin and eosin (HE) stained sections under an optical microscope. Tumor cells in the form of micropapillary clusters, solid nests, or single cells were identified in lung tissues situated more than three alveolar intervals away from the main tumor boundary. In instances of disagreement, consensus was reached through discussion. The classification of lung cancer adhered to the WHO thoracic tumor classification standard (2021 edition) (Nicholson et al. 2022), and clinical staging was determined according to the Tumor, Node, and Metastasis (TNM) staging criteria (8th edition) for lung cancer (Travis et al. 2016).

### Statistical analysis

Statistical analysis was conducted using SPSS 20.0 software and R software (R version 4.2.2). The normality of measurement data was assessed using the Shapiro–Wilk test. Measurement data following a normal distribution were presented as x̄ ± s. Group comparisons were made using the independent sample *t* test. Measurement data not conforming to a normal distribution were expressed as M (Q1, Q3), and group comparisons were conducted using the Mann–Whitney U test. Categorical data were presented as the number of cases, and inter-group comparisons were performed using Pearson’s Chi-square test, Yate’s correction for continuity, or Fisher’s exact test. In univariable analysis, a significance level of *P* < 0.05 was considered statistically significant. For the training set, variables with *P* < 0.1 in univariable analysis were included in multivariable logistic regression analysis. Based on the minimization of Akaike information criterion (AIC) (Portet et al. 2020), the optimal combination of predictors was selected to construct the model and the corresponding nomogram was developed. The receiver operating characteristic (ROC) curve and AUC value were used to evaluate the model’s differentiation in the training and external testing set. The DeLong test was used to assess differences in AUC between models. Calibration curves and Hosmer–Lemeshow (H–L) test were used to assess the goodness-of-fit of the model, and the P value greater than 0.05 indicated a well goodness-of-fit. Decision curve analysis (DCA) was used to assess the clinical application value of the model. Kappa coefficient and intraclass correlation coefficient (ICC) were utilized to evaluate the consistency of qualitative and quantitative parameters among observers, with *P* < 0.05 considered statistically significant.

## Results

### Comparison of clinicopathological data

Among the 180 patients diagnosed with NSCLC, 152 cases were identified as LUAD, 24 cases as lung squamous cell carcinoma, 2 cases as lung adenosquamous carcinoma, and 2 cases as large cell lung cancer. In the training set, 45 cases were STAS-positive (38.8%) and 71 cases were STAS-negative (61.2%). In the testing set, 33 cases were STAS-positive (51.6%) and 31 cases were STAS-negative (48.4%). Statistical analysis revealed significant differences in pathological types in the training set (P < 0.05) (Table 1). Regarding CT features, the quantitative parameters showed good consistency between the two observers, with ICC values ranging from 0.960 to 1.000. In addition, the qualitative indicators exhibited strong consistency, with Kappa values ranging from 0.881 to 1.000 (See Supplemental Table 2).

**Table 2:** CT features and PET parameters of patients in the training and testing set

Variables	Training set (*n* = 116)			Testing set (*n* = 64)		
	STAS (-) (*n* = 71)	STAS ( +) (*n* = 45)	*P* value	STAS (-) (*n* = 31)	STAS ( +) (*n* = 33)	*P* value
SUV_max_	1.9 (1.2,4.5)	5.6 (3.8,10.1)	< 0.001^c^	2.9 (1.5,6.8)	10.1 (6.1,13.9)	< 0.001^c^
Tumor diameter	2.1 (1.5,2.6)	2.4 (1.8,2.7)	0.055^c^	2.1 ± 0.6	2.4 ± 0.4	0.027^d^
Solid component diameter	1.4 (1.0,1.9)	2.3 (1.7,2.7)	< 0.001^c^	1.6 ± 0.6	2.3 ± 0.5	< 0.001^d^
CTR	0.7 (0.5,1.0)	1.0 (1.0,1.0)	< 0.001^c^	1.0 (0.7,1.0)	1.0 (1.0,1.0)	0.012^c^
Lymph node uptake			0.143^a^			0.355^b^
Absent	56 (78.9%)	30 (66.7%)		28 (90.3%)	26 (78.8%)	
Present	15 (21.1%)	15 (33.3%)		3 (9.7%)	7 (21.2%)	
Density type			< 0.001^a^			0.033^a^
Part-solid	38 (53.5%)	8 (17.8%)		12 (38.7%)	5 (15.2%)	
Solid	33 (46.5%)	37 (82.2%)		19 (61.3%)	28 (84.8%)	
Interface			0.476^a^			0.510^a^
Well-defined	51 (71.8%)	35 (77.8%)		23 (74.2%)	22 (66.7%)	
Ill-defined	20 (28.2%)	10 (22.2%)		8 (25.8%)	11 (33.3%)	
Lobulation			0.012^a^			0.024^b^
Absent	20 (28.2%)	4 (8.9%)		8 (25.8%)	1 (3.0%)	
Present	51 (71.8%)	41 (91.1%)		23 (74.2%)	32 (97.0%)	
Spiculation			0.004^a^			0.313^a^
Absent	52 (73.2%)	21 (46.7%)		18 (58.1%)	15 (45.5%)	
Present	19 (26.8%)	24 (53.3%)		13 (41.9%)	18 (54.5%)	
Bronchial change			0.916^a^			0.876^a^
Absent	23 (32.4%)	15 (33.3%)		9 (29.0%)	9 (27.3%)	
Present	48 (67.6%)	30 (66.7%)		22 (71.0%)	24 (72.7%)	
Pleural indentation			0.224^a^			0.136^a^
Absent	35 (49.3%)	17 (37.8%)		14 (45.2%)	9 (27.3%)	
Present	36 (50.7%)	28 (62.2%)		17 (54.8%)	24 (72.7%)	
Vascular convergence			0.004^a^			0.264^a^
Absent	52 (73.2%)	21 (46.7%)		22 (71.0%)	19 (57.6%)	
Present	19 (26.8%)	24 (53.3%)		9 (29.0%)	14 (42.4%)	
Emphysema background			0.071^a^			0.123^a^
Absent	65 (91.5%)	36 (80.0%)		28 (90.3%)	25 (75.8%)	
Present	6 (8.5%)	9 (20.0%)		3 (9.7%)	8 (24.2%)	
Ground glass ribbon sign			0.031^a^			0.023^a^
Absent	46 (64.8%)	20 (44.4%)		21 (67.7%)	13 (39.4%)	
Present	25 (35.2%)	25 (55.6%)		10 (32.3%)	20 (60.6%)	

Note: a, Pearson’s Chi-square; b, Yate’s correction for continuity; c, the Mann–Whitney U test; d, the two independent samples *t* test; STAS, spread through air spaces; SUV_max_, the maximum standardized uptake value; CTR, consolidation-to-tumor ratio

### Univariable and multivariable logistic regression analysis

**Fig. 2:**
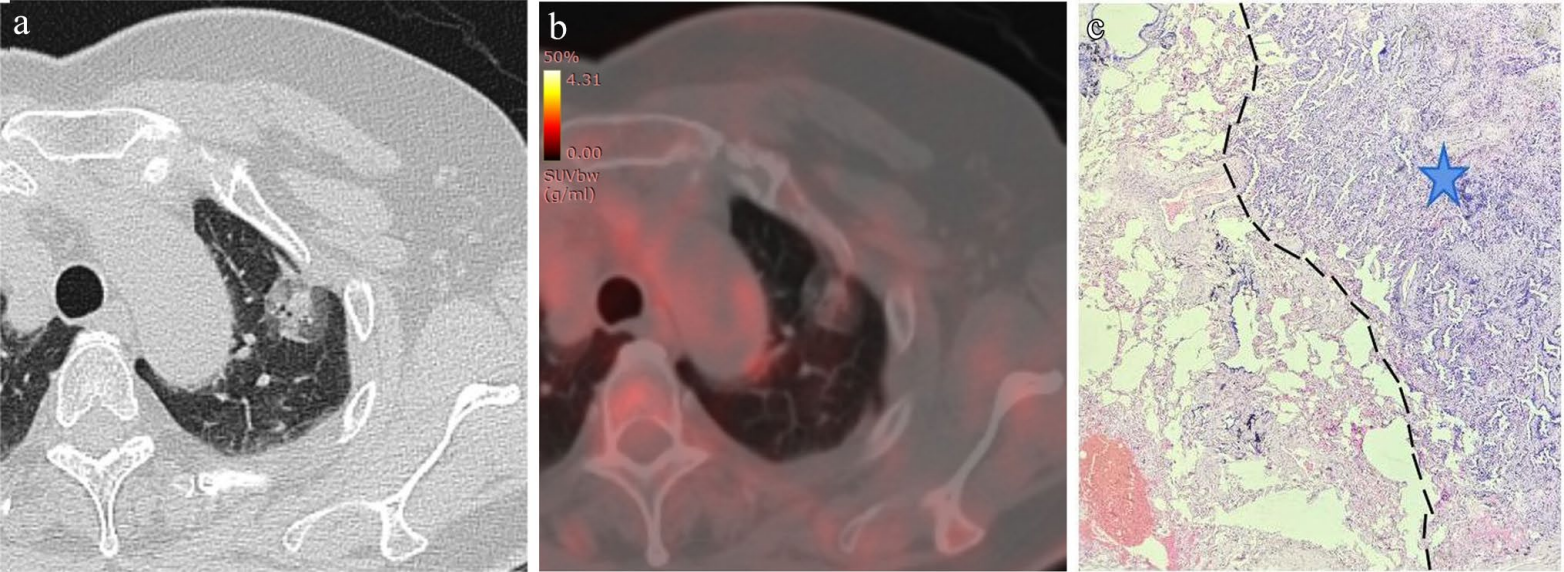
A 66-year-old female patient with LUAD and negative STAS status. **A** The HRCT shows a part-solid nodule in the left upper lobe of the lung with the longest diameter of the whole tumor and solid component as 2.5 cm and 1.2 cm, respectively. **B** The.^18^F-FDG PET/CT shows that the SUV_max_ of the nodule is 0.72. (2C) Photomicrograph (hematoxylin–eosin stain, magnification 200) shows clean alveolar spaces adjacent to boundary of the tumor ( +)

**Fig. 3:**
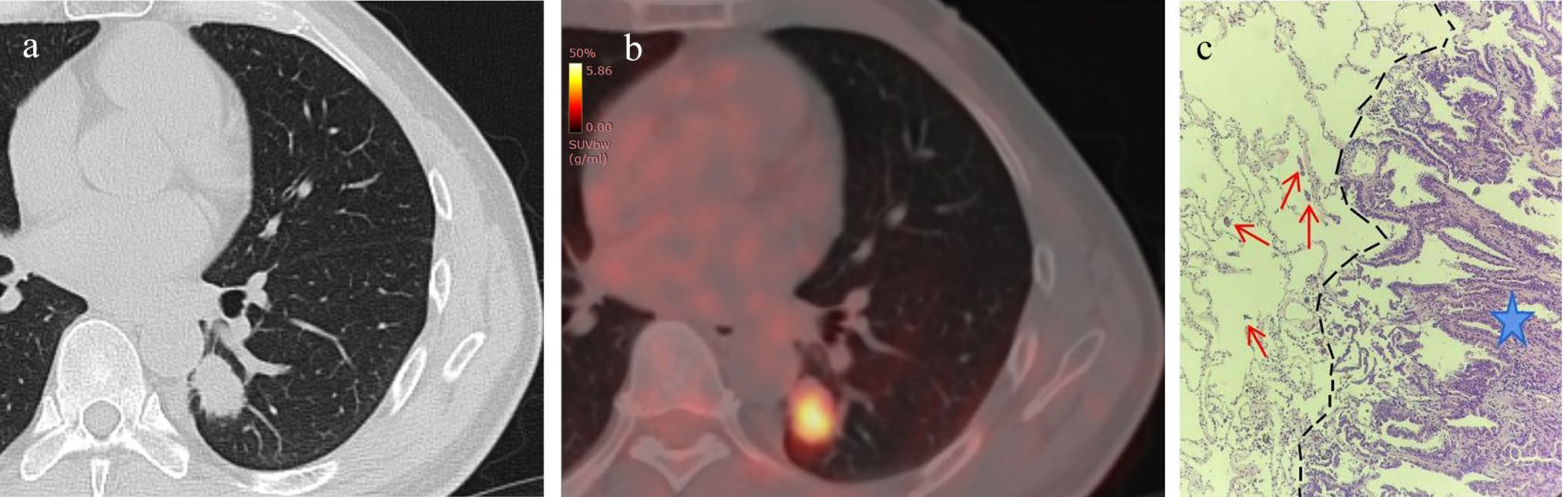
A 51-year-old male patient with LUAD and positive STAS status. **A** The HRCT shows a solid nodule in the left lower lobe of the lung with the longest diameter of the whole tumor as 2.6 cm. **B** The ^18^F-FDG PET/CT shows that the SUVmax of the nodule is 5.3. **C** Photomicrograph (Hematoxylin–eosin stain, magnification 200) shows detached micropapillary clusters of tumor cells (red arrows) in alveolar beyond the edge of the main tumor ( +)

In the training set, the univariable analysis revealed significant differences in SUVmax, tumor diameter, solid component diameter, CTR, density type, lobulation, spiculation, vascular convergence, and Ground glass ribbon sign between the STAS-positive group and the STAS-negative group (*P* < 0.05) (Table 2). Variables with *P* < 0.1 in the univariable analysis were included in the multivariable logistic regression analysis. The results from the multivariable logistic regression analysis identified SUV_max_, CTR, and lobulation sign as the best predictive variables. SUV_max_ (OR = 1.206) and CTR (OR = 15.92) were identified as independent risk predictors for predicting STAS in NSCLC (Table 3). Subgroup analysis within solid nodular NSCLC (*n* = 70) showed statistically significant differences in SUV_max_, tumor diameter, and solid component diameter between the STAS-positive and STAS-negative groups (*P* < 0.05). SUV_max_ (OR = 1.229) was identified as an independent risk predictor for predicting STAS in solid nodular NSCLC. In part-solid nodular NSCLC (*n* = 46), solid component diameter and CTR were significantly different between the STAS-positive and STAS-negative groups (*P* < 0.05). CTR (OR = 7.134) and emphysema background (OR = 2.596) were identified as independent risk predictors for predicting STAS (Table 4). Typical ^18^F-FDG PET/CT case images are illustrated in Figs. 2 and 3.

**Table 3:** Univariable and multivariable logistic regression analysis of predictors in the training set

Predictors	Univariable logistic regression analysis		Multivariable logistic regression analysis	
	OR (95% CI)	*P* value	OR (95% CI)	*P* value
SUV_max_	1.308 (1.166–1.497)	< 0.001	1.206 (1.067–1.393)	0.005
Tumor diameter	1.905 (1.032–3.638)	0.044		
Solid component diameter	3.571 (2.003–6.780)	< 0.001		
CTR	79.49 (10.30–920.5)	< 0.001	15.92 (1.516–223.8)	0.027
Density type	5.326(2.262–13.79)	< 0.001		
Lobulation	4.020(1.390–14.64)	0.018	3.035(0.926–12.04)	0.083
Spiculation	3.128 (1.437–6.971)	0.005		
Vascular convergence	3.128 (1.437–6.971)	0.005		
Emphysema background	2.708 (0.904–8.662)	0.079		
Ground glass ribbon sign	2.300 (1.078–4.991)	0.033		

Note: OR, odds ratio; CI, confidence interval; SUV_max_, the maximum standardized uptake value; CTR, consolidation-to-tumor ratio

**Table 4:** Univariable and multivariable logistic regression analysis of solid and part-solid nodules in the training set

Predictors	Univariable logistic regression analysis		Multivariable logistic regression analysis	
Solid nodules	OR (95% CI)	*P* value	OR (95% CI)	*P* value
SUV_max_	1.229 (1.077–1.445)	0.006	1.229 (1.077–1.445)	0.005
Tumor diameter	2.230 (1.040–5.065)	0.045		
Solid component diameter	2.230 (1.040–5.065)	0.045		
Vascular convergence	2.353 (0.904–6.363)	0.084		
Part-solid nodules				
Solid component diameter	5.255 (0.942–44.05)	0.080		
CTR	12.63 (3.651–205.7)	0.029	7.134 (4.702–9.520)	0.019
Emphysema background	12.33 (1.031–291.6)	0.054	2.596 (1.659–8.121)	0.028

Note: OR, odds ratio; CI, confidence interval; SUV_max_, the maximum standardized uptake value; CTR, consolidation-to-tumor ratio

### Construction and efficiency analysis of the prediction model

**Fig. 4:**
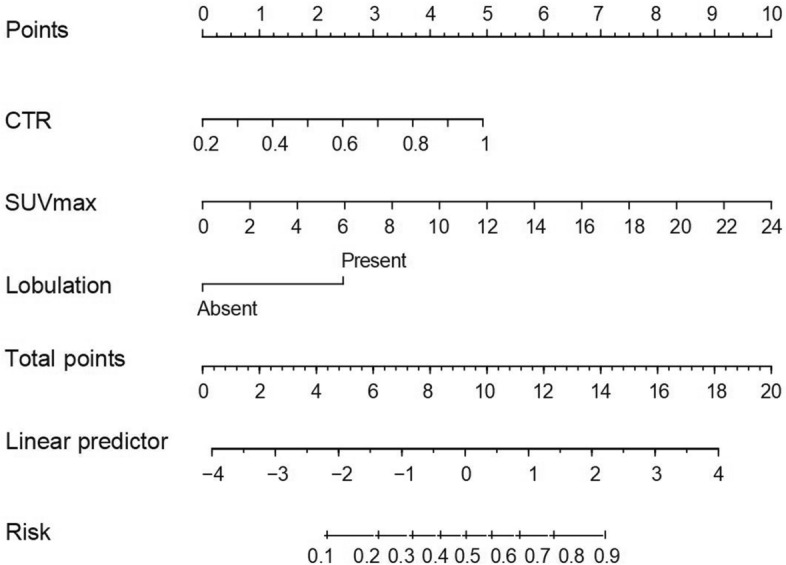
Nomogram for preoperative prediction of STAS status based on ^18^F-FDG PET/CT radiological features and SUV_max_ in clinical-stage IA NSCLC

**Fig. 5:**
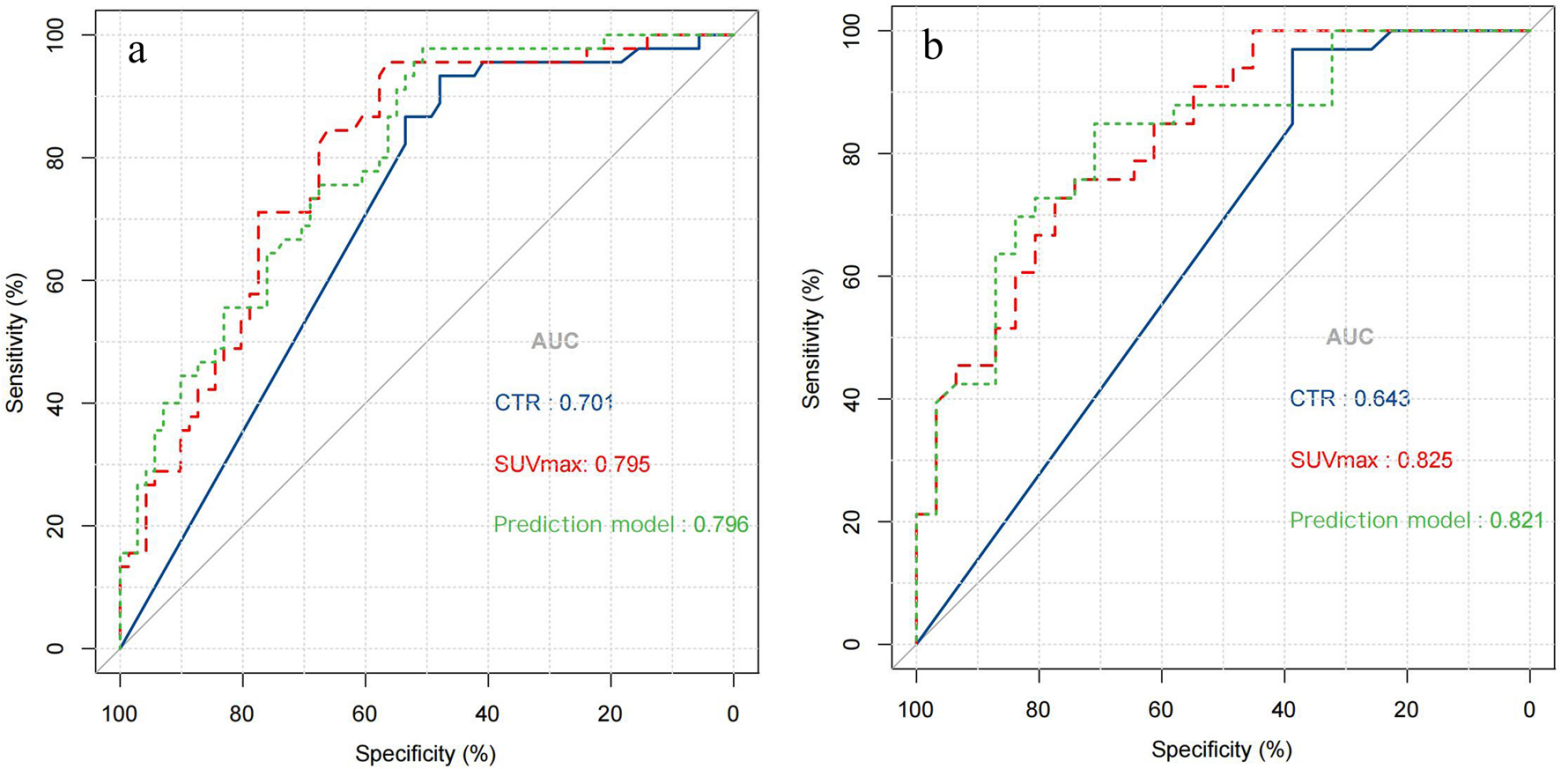
ROC curve analysis of CTR, SUV_max_, and the prediction model in the training and testing set

**Fig. 6:**
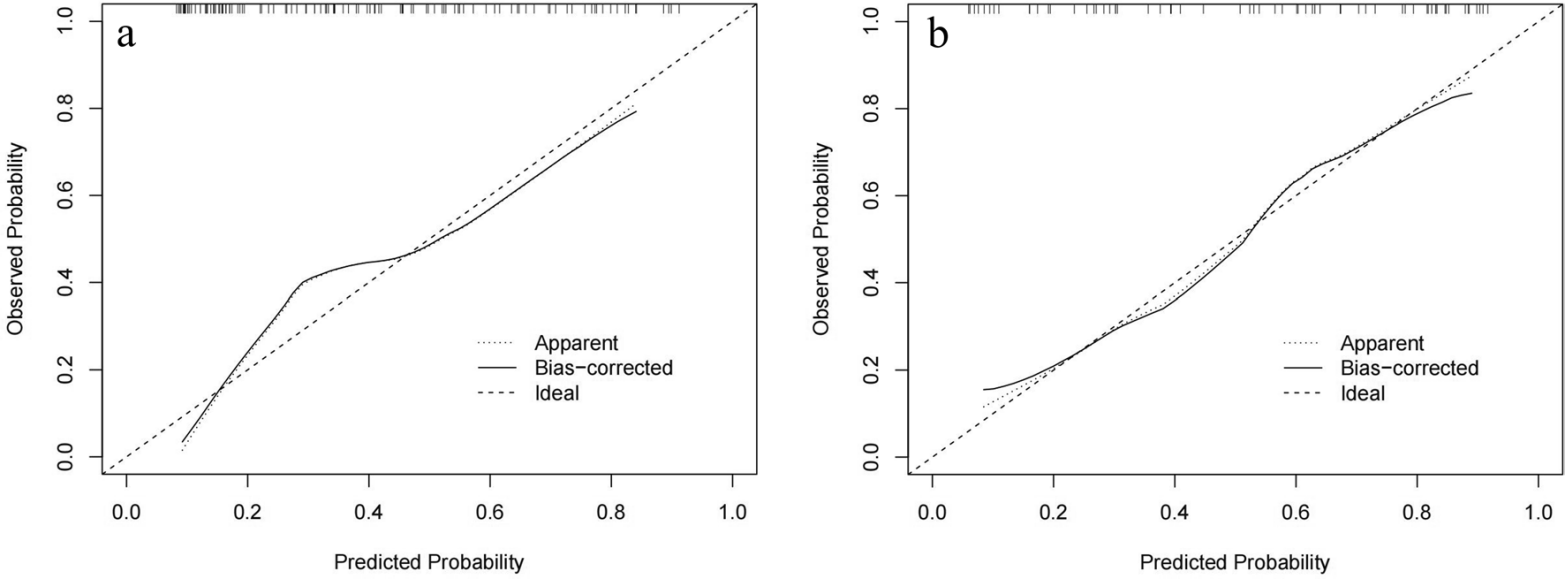
The calibration curves of the prediction model in the training and testing set

**Fig. 7:**
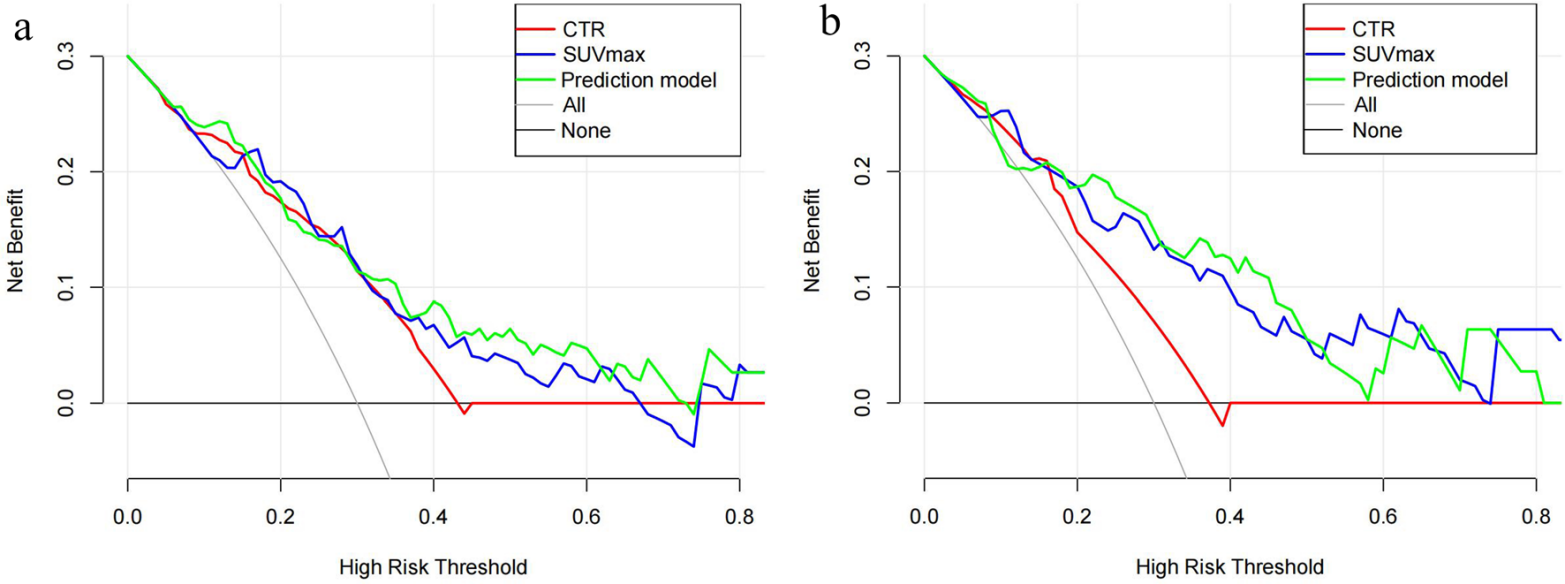
The decision curves of CTR, SUV_max_, and the prediction model in the training and testing set

Based on the best combination of the variables identified through multivariable logistic regression analysis, a prediction model was constructed, and a nomogram was generated (Fig. 4). The regression equation was ln(P/1-P) = -4.702 + 2.768 × CTR + 0.187 × SUV_max_ + 1.110 × lobulation, where P is the probability of predicting STAS occurrence. The predictive probability value and single predictors of the multivariable logistic regression model were analyzed by the ROC curve. In the training set, the AUC values for CTR, SUV_max_, and the prediction model in predicting STAS were 0.701, 0.795, and 0.796, with accuracies of 65.52%, 71.55%, and 68.97%, respectively. In the testing set, the AUC values were 0.643, 0.825, and 0.821, respectively, with accuracies of 68.75%, 75.00%, and 78.13% (Table 5). The ROC curve is presented in Fig. 5. The DeLong test indicated that the prediction model was superior to CTR (Z = 3.181, 3.801, *P* < 0.05), and SUV_max_ was better than CTR (Z = 2.411, 3.105, *P* < 0.05). The H–L test demonstrated a good fit for the prediction model in both the training set and the testing set (*P* = 0.12, 0.36) (Fig. 6). DCA illustrated that the prediction model, SUV_max_, and CTR have good clinical application value (Fig. 7).

**Table 5:** The predictive efficacy of CTR, SUV_max_, and prediction model in the training and testing set

	Set	Cut-off	AUC (95% CI)	Accuracy (%)	Sensitivity (%)	Specificity (%)	PPV (%)	NPV (%)
CTR	Training	0.66	0.701 (0.609–0.783)	65.52%	93.33%	47.89%	53.16%	91.89%
	Testing		0.643 (0.513–0.759)	68.75%	96.97%	38.71%	62.75%	92.31%
SUV_max_	Training	2.08	0.795 (0.710–0.864)	71.55%	95.56%	56.34%	58.11%	95.24%
	Testing		0.825 (0.709–0.908)	75.00%	72.73%	77.42%	77.42%	72.73%
Prediction model	Training	0.189	0.796 (0.711–0.865)	68.97%	97.78%	50.70%	55.70%	97.30%
	Testing		0.821 (0.705–0.905)	78.13%	84.85%	70.97%	75.68%	81.48%

Note: AUC, area under the curve; CI, confidence interval; PPV, positive predictive value; NPV, negative predictive value; CTR, consolidation-to-tumor ratio; SUV_max_, the maximum standardized uptake value

## Discussion

STAS is a crucial invasive pattern in lung cancer that significantly impacts postoperative tumor recurrence and metastasis. The accurate preoperative assessment of STAS status is essential for effectively stratifying patient risks, guiding surgical method selection, and improving overall survival prognosis. This study identified CTR and SUV_max_ as independent predictors for STAS. The constructed prediction model, incorporating CTR, SUV_max_, and lobulation, demonstrated excellent diagnostic efficacy with AUC values of 0.796 in the training set and 0.821 in the testing set. Overall, our nomogram provides a simple and efficient tool for predicting STAS status, offering valuable guidance for clinical treatment decision-making.

In the present study, lobulation, spiculation, vascular convergence, and the Ground glass ribbon sign were more prevalent in the STAS-positive group, aligning with findings in previous literature (Chen et al. 2022; Qi et al. 2021; Toyokawa et al. 2018). Lobulation reflects the expansive growth of the tumor, indicating varying growth rates of cells around the tumor. Spiculation is indicative of tumor tissue invasion into adjacent structures, suggesting increased tumor aggressiveness (Jin et al. 2017). Vascular convergence sign results from tumor infiltration and growth into adjacent pulmonary blood vessels and bronchial bundles, with increased fibrous hyperplasia pulling together pulmonary blood vessels (Chen et al. 2022; Zhang et al. 2014), suggesting a greater risk of STAS. The Ground glass ribbon sign, a ribbon shadow extending from the nodule’s edge, is speculated to occur when tumor cells escape from the primary lesion, redistribute through the airway, and grow along the surrounding alveolar wall. This leads to the obstruction of surrounding lung parenchyma or bronchioles, resulting in reduced gas content in the alveoli. The presence of the Ground glass ribbon sign was more common in the STAS-positive group, consistent with previous studies (Qi et al. 2021). While the evaluation of CT features is subjective, and previous studies have reached different conclusions, the overall trend suggests that more malignant tumor characteristics increase the likelihood of STAS positivity.

This study observed a positive correlation between tumor diameter, solid component diameter, and CTR with STAS status. The incidence of STAS in solid nodular NSCLC was 2.69 times higher than that in part-solid nodular NSCLC. Multivariable logistic regression analysis confirmed that CTR was an independent risk predictor for predicting STAS, consistent with prior literature reports (Kim et al. 2018; Chen et al. 2022; Qi et al. 2021; Li et al. 2020; Zhang et al. 2020). Compared to tumor diameter and solid component diameter, CTR better represents the more aggressive part of the tumor. The higher the proportion of CTR, the greater the positive rate of STAS, aligning with clinical T staging based on the diameter of the solid component rather than the overall tumor diameter (Travis et al. 2016). Subgroup analysis revealed that CTR and emphysema background were independent risk predictors for predicting STAS in part-solid nodular NSCLC, while SUV_max_ was not a risk predictor for predicting STAS status. It is widely thought that ground glass components in part-solid nodules exhibit slower growth, with fewer tumor cells in this area, resulting in lower maximum FDG uptake than in solid nodules (Travis et al. 2016). This study suggests that CT features should be emphasized when predicting STAS in part-solid nodular NSCLC.

As the most commonly used semi-quantitative index reflecting the glucose metabolic activity of tumor cells, SUV_max_ can be employed to assess the biological malignancy of tumor cells (de Geus-Oei et al. 2007). In this study, SUV_max_ emerged as an independent risk predictor for predicting STAS. Higher SUV_max_ values indicate stronger metabolic activity and tumor aggressiveness, correlating with an increased positive rate of STAS, consistent with findings from prior studies (Wang et al. 2020; Nishimori et al. 2022). Subgroup analysis revealed that SUV_max_ was an independent risk predictor for predicting STAS in solid nodular NSCLC. These results underscore the importance of careful consideration for the possibility of STAS in clinical-stage IA NSCLC cases with solid nodules on ^18^F-FDG PET/CT, particularly in instances of elevated SUV_max_.

This study focused on clinical-stage IA NSCLC, integrating ^18^F-FDG PET/CT metabolic parameters and CT characteristics associated with STAS. By constructing a nomogram, the intricate regression equation was transformed into a user-friendly graphical tool, aiding in the preoperative assessment of STAS status and assisting in the development of clinical diagnosis plans for patients. In addition, the generalization of the model was further validated using external datasets in this study. The DeLong test demonstrated that the prediction model and SUV_max_ exhibited superior diagnostic efficacy compared to CTR (P < 0.05). This suggests that CTR may have certain limitations in predicting STAS, speculating that solid components in tumors may result from fibroblast proliferation, alveolar collapse, inflammatory cell infiltration, and mucous secretion by tumor cells (Park et al. 2007). CTR may not entirely capture the invasiveness of the tumor. On the other hand, SUV_max_, as an indicator reflecting the FDG metabolism of tumors, represents the proliferative activity of tumor cells and better reflects tumor aggressiveness. In general, a higher SUV_max_ value corresponds to a greater risk of STAS.

This study has several limitations. First, it is a retrospective study, and a certain degree of selection bias is inevitable. Second, the external testing set data in this study are relatively small, and larger multi-center prospective studies are needed to validate the conclusions in the future. Third, with the current focus on radiomics and deep learning, the next steps should involve building a multi-modal deep learning model based on convolutional neural networks, incorporating ^18^F-FDG PET/CT metabolic parameters, CT features, clinical data, and radiomics features, aiming to assess whether such a model can enhance the diagnostic efficiency of predicting STAS. Fourth, due to the lack of detailed long-term follow-up data, whether STAS is an independent prognostic factor and the effect of STAS on patients undergoing sublobectomy should be further confirmed, we will conduct this study in the future when more follow-up data are collected.

## Conclusion

In conclusion, STAS in clinical-stage IA NSCLC exhibits a strong correlation with ^18^F-FDG PET/CT parameter information. The nomogram constructed by incorporating SUV_max_, CTR, and lobulation sign proves to be a valuable tool for predicting STAS status, offering guidance for clinical diagnosis and treatment decisions. Furthermore, SUV_max_ emerges as an independent risk predictor for predicting STAS in solid nodules. For part-solid nodules, prioritizing CTR and assessing emphysema background is recommended. The careful observation of these CT features contributes to enhancing the accuracy of predicting STAS status preoperatively.

### Supplementary Information

Below is the link to the electronic supplementary material.

Supplementary file 1: (DOCX 1311 KB)

## Data Availability

The raw data supporting the conclusions of this article will be made available by the authors, without undue reservation.
