# Supplementary material for: Preoperative nomogram for predicting spread through air spaces in clinical-stage IA non-small cell lung cancer using 18F-fluorodeoxyglucose positron emission tomography/computed tomography
Source: J Cancer Res Clin Oncol. 2024 Apr 10;150(4):185. doi: 10.1007/s00432-024-05674-w (PMC11006761; doi:10.1007/s00432-024-05674-w)
Supplement: Supplementary file 1 — (DOCX 1311 KB) [file 432_2024_5674_MOESM1_ESM.docx]

**Supplemental Content 1**

Training set：

Whole-body PET/CT tomography was performed using a Siemens Biograph Truepoint 64 PET/CT (Germany). The fasting blood glucose level of the patient was less than 10 mmol/L before the examination. After measuring the patient’s body weight, the patient was given an intravenous injection of ^18^F-FDG (Shanghai Atomic Kexing Pharmaceutical Co, Ltd.) 3.70–5.55 MBq/kg (0.10–0.15 mCi/kg) body weight with a radiochemical purity >95%, followed by a 300 mL water drink.The patient was instructed to lie down and rest for 60 min in a dark room. After emptying the bladder, the body scan ranged from the base of the skull to the middle of the femur, scanning 5–7 beds, 2–3 min/bed, with a reconstruction matrix of 192 × 192. The PET images were attenuated by CT images and reconstructed iteratively. The body CT scanning parameters were as follows: tube voltage 120 kV, tube current 160 mAs, scanning layer thickness 3.75 mm, reconstruction matrix 512 × 512, and pitch 0.8 s. The chest high-resolution computed tomography (HRCT) scan parameters were as follows: tube voltage 120 kV, tube current 150 mAs, scanning layer thickness 5 mm, reconstruction layer thickness and layer interval 1 mm, reconstruction matrix 512 × 512, and pitch 0.8 s. The lung window images were reconstructed by a high-resolution algorithm (B70f), and the mediastinal window images were reconstructed using a standard algorithm (B40f).

Testing set：

Prior to the ^18^F-FDG PET/CT scan, study participants were asked to fast for at least 6 h, ensuring a blood glucose (BG) less than 11.1 mmol/L, and then they were intravenously administered ^18^F-FDG (3.70–5.55 MBq/kg). All acquisitions were performed on a Biograph 64 PET/CT scanner (Siemens Healthcare, Erlangen, Germany) 45–60 min after ^18^F-FDG injection. The whole-body CT scanning parameters were set as follows: current (170 mA), voltage (120 kV), and scan layer thickness (3 mm). The PET scan was performed after CT scan acquisition and conducted in 5–6 bed positions. Immediately after CT image acquisition, PET data were acquired for 3 min per bed position. Reconstruction of the acquired data was performed by the postprocessing workstation with an iterative TrueD reconstruction system (Siemens Medical Solutions). Correction attenuation was performed by CT images. HRCT scan parameters were as follows: tube voltage 120 kV, tube current 100 mAs.

**Supplemental Table 1** The definitions of CT features

| Features | Definition |
| --- | --- |
| Tumor diameter | The longest diameter of the whole tumor at the lung window on the MPR image |
| Solid component diameter | The longest diameter of the solid component of the tumor at the lung window on the MPR image |
| CTR | The proportion of the solid component part (consolidation-to-tumor ratio, CTR) |
| Lymph node uptake | Lymph node uptake was higher than liver, and postoperative pathology showed no pathological lymph nodes |
| Density type | Part-solid, presence of ground-glass opacity and solid density component; solid, absence of ground-glass opacity, contains solid density component only |
| Interface | Interface of the tumor-lung, including well-defined or ill-defined interface |
| Lobulation | Petaloid or wavy appearance at the tumor’s margins |
| Spiculation | Short, thin linear strands radiating around the surface of the tumor without reaching the pleural surface |
| Bronchial change | Air-filled bronchus manifesting as natural, dilated/distorted or cut-off within the lesions, or cut-off at the edge of the lesions |
| Pleural indentation | The deviation of the pleura from its original position due to tumor traction at the lung window |
| Vascular convergence | The convergence of pulmonary vessels around the tumor towards the lesion |
| Emphysema background | Presence of emphysema in the lobe of lung cancer with visual observation |
| Distal ribbon sign | A long, thick ribbon strand extending from the distal part of the tumor into the surrounding lung tissue |

**Supplemental Table 2** Consistency analysis of CT features among observers

|  | Kappa (95% CI) |  |  | ICC (95% CI) |
| --- | --- | --- | --- | --- |
| Density type | 0.968 (0.939-0.996) |  | SUVmax | 1.000 |
| Interface | 0.881 (0.800-0.962) |  | Tumor diameter (cm) | 0.960 (0.933-0.987) |
| Lobulation | 0.953 (0.916-0.990) |  | Solid component diameter (cm) | 0.963 (0.934-0.992) |
| Spiculation | 0.925 (0.859-0.990) |  |  |  |
| Bronchial change | 0.970 (0.947-0.994) |  |  |  |
| Pleural indentation | 0.934 (0.911-0.950) |  |  |  |
| Vascular convergence | 0.887 (0.834-0.941) |  |  |  |
| Distal ribbon sign | 0.935 (0.919-0.947) |  |  |  |
| Lymph node uptake | 1.000 |  |  |  |
| Emphysema background | 1.000 |  |  |  |

**Supplementary Figures**


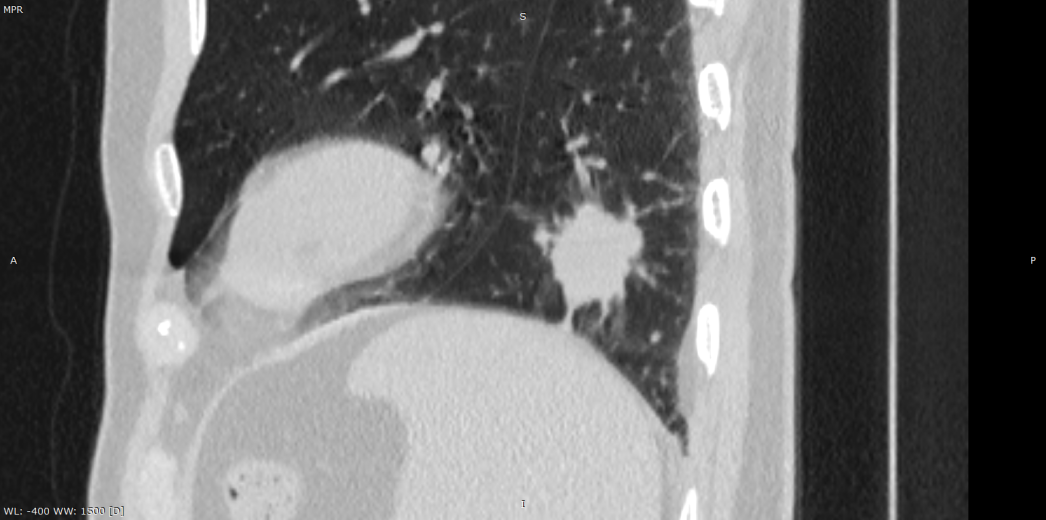

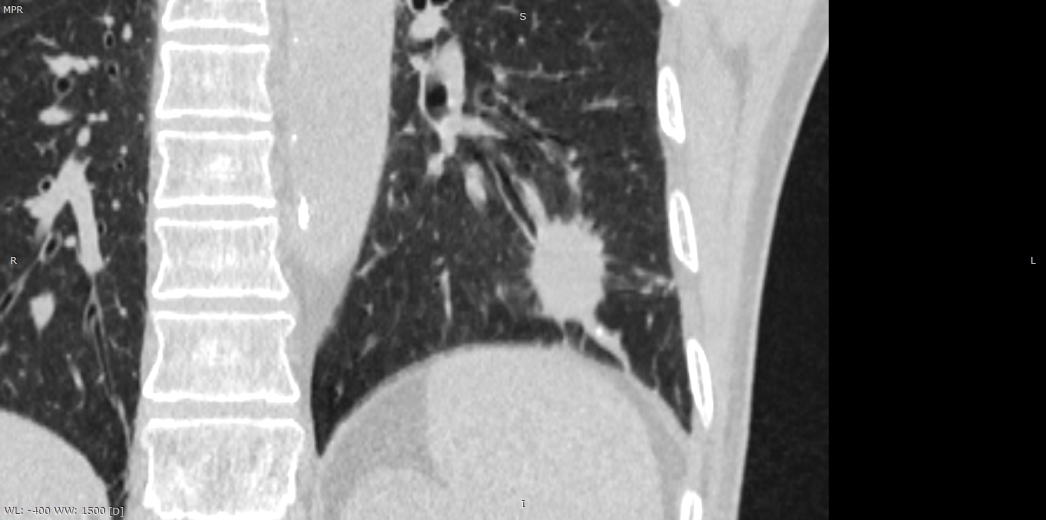


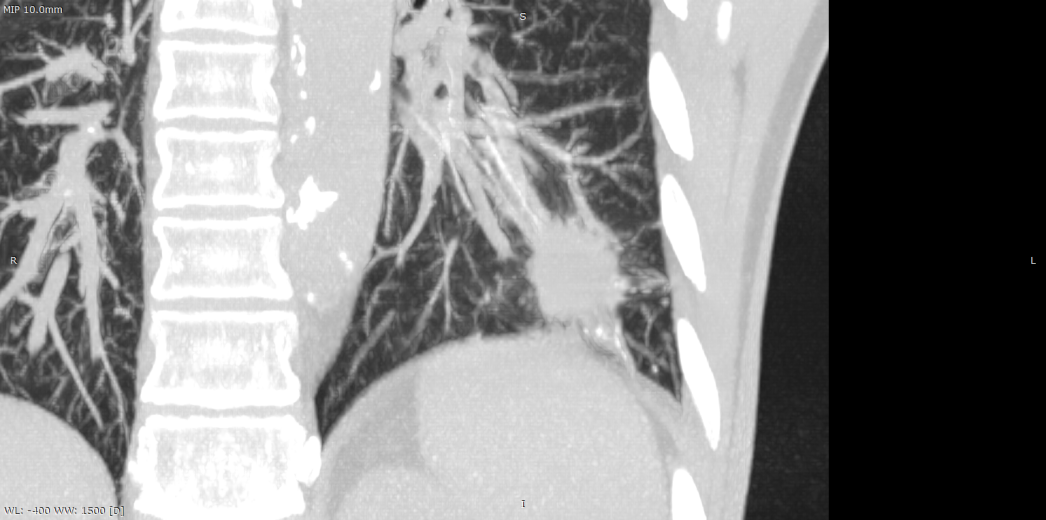


**Figure S1 to Figure S3** show a same patient, a 75-year-old male patient with lung squamous cell carcinoma and positive STAS status. The axial non-contrast computed tomography shows a solid nodule in the left lower lobe of the lung, with lobulation sigh (Figure S1, blue arrow), distal ribbon sign (Figure S2, purple arrow) , bronchial change (Figure S2, yellow arrow). The maximal intensity projection shows a vascular convergence sign (Figure S3,black arrows).


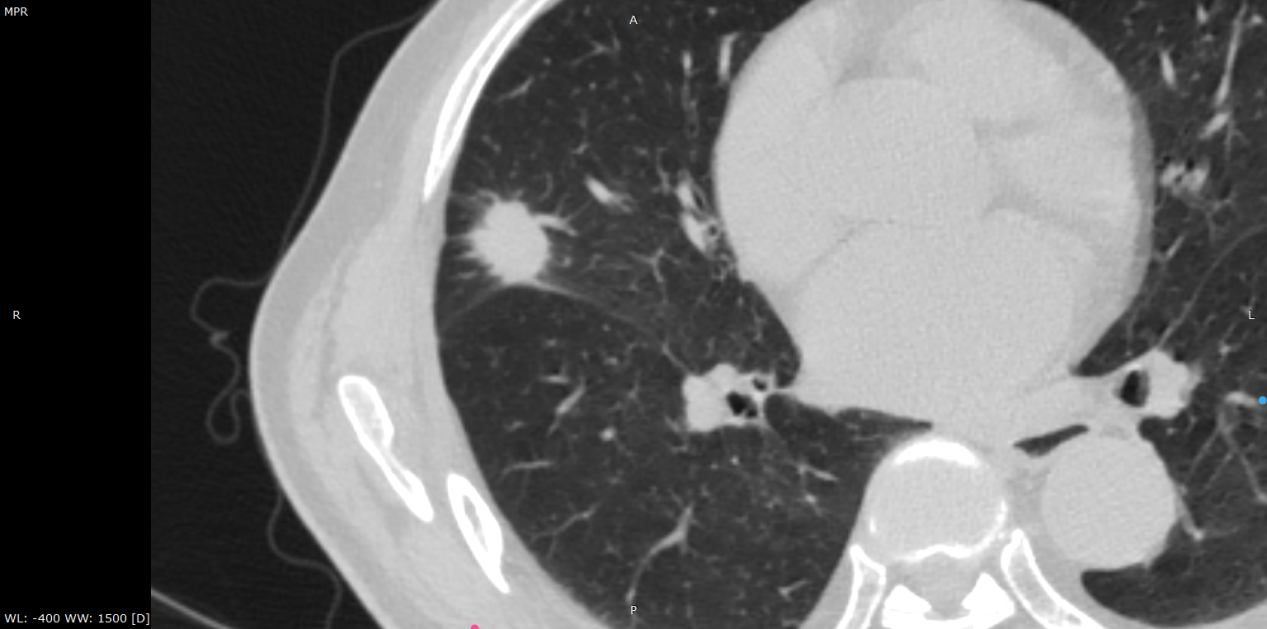


**Figure S4** show a 78-year-old male patient with lung squamous cell carcinoma and positive STAS status. The axial non-contrast computed tomography shows a solid nodule in the right middle lobe of the lung, with spiculation sign (Figure S4, red arrow), pleural indentation sigh (Figure S4, green arrow).


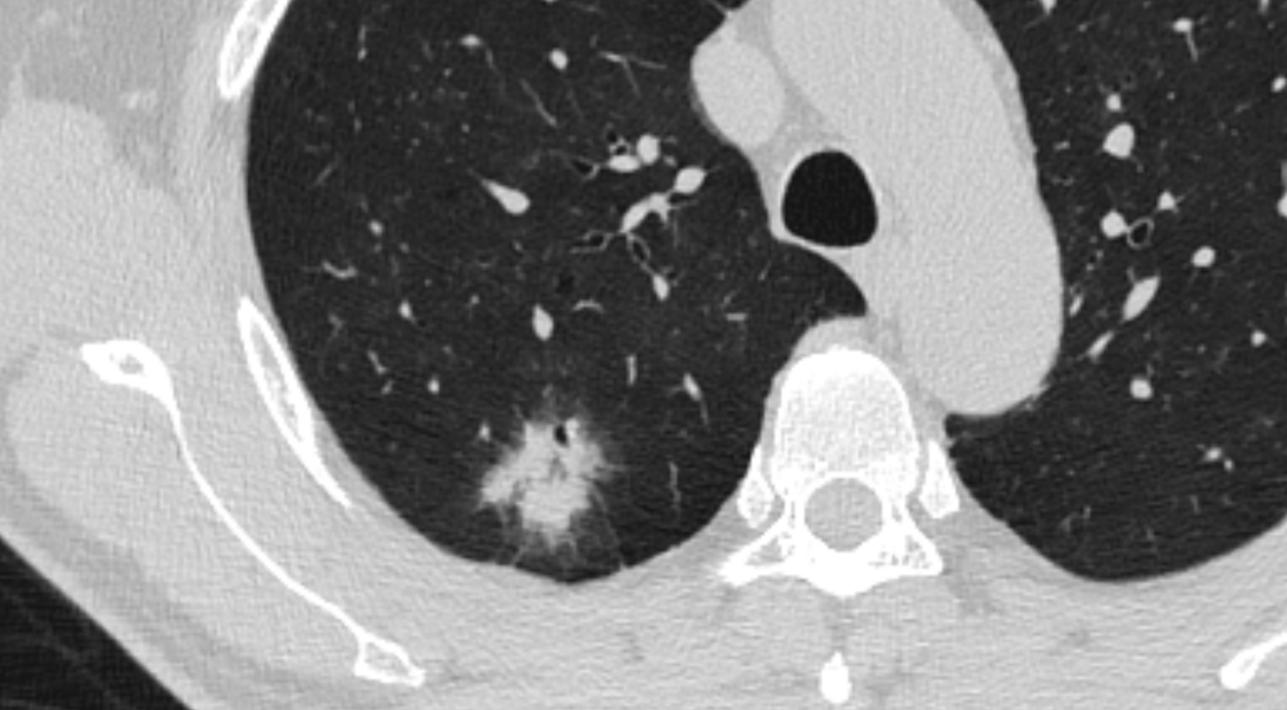


**Figure S5** show a 50-year-old male patient with lung adenocarcinoma and positive STAS status. The axial non-contrast computed tomography shows a part-solid nodule in the right upper lobe of the lung, with emphysema background (Figure S5, blue arrow).
